# Broad Range of Hepatitis B Virus (HBV) Patterns, Dual Circulation of Quasi-Subgenotype A3 and HBV/E and Heterogeneous HBV Mutations in HIV-Positive Patients in Gabon

**DOI:** 10.1371/journal.pone.0143869

**Published:** 2016-01-14

**Authors:** Berthold Bivigou-Mboumba, Sandrine François-Souquière, Luc Deleplancque, Jeanne Sica, Augustin Mouinga-Ondémé, Marie Amougou-Atsama, Marie-Laure Chaix, Richard Njouom, François Rouet

**Affiliations:** 1 Laboratoire de Rétrovirologie, Centre International de Recherches Médicales de Franceville (CIRMF), Franceville, Gabon; 2 Unité Mixte de Recherche VIH et Maladies Infectieuses Associées (UMR-VIH-MIA), CIRMF, Libreville, Gabon; 3 Centre de Traitement Ambulatoire (CTA), Franceville, Gabon; 4 Service de Virologie, Centre Pasteur du Cameroun, Yaoundé, Cameroun; 5 Laboratoire de Virologie, AP-HP, Hôpital Saint Louis; INSERM U941, Université Paris Diderot; Laboratoire associé au Centre national de Référence du VIH, Paris, France; CEA, FRANCE

## Abstract

Integrated data on hepatitis B virus (HBV) patterns, HBV genotypes and mutations are lacking in human immunodeficiency virus type 1 (HIV-1) co-infected patients from Africa. This survey was conducted in 2010–2013 among 762 HIV-1-positive adults from Gabon who were predominantly treated with 3TC-based antiretroviral treatment. HBV patterns were identified using immunoassays detecting total antibody to hepatitis B core antigen (HBcAb), hepatitis B surface antigen (HBsAg), IgM HBcAb, hepatitis B e antigen (HBeAg), antibody to HBsAg (HBsAb) and an in-house real-time PCR test for HBV DNA quantification. Occult hepatitis B (OBI) was defined by the presence of isolated anti-HBc with detectable serum HBV DNA. HBV genotypes and HBV mutations were analyzed by PCR-direct sequencing method. Seventy-one (9.3%) patients tested positive for HBsAg, including one with acute hepatitis B (0.1%; 95% CI, 0.0%-0.2%), nine with HBeAg-positive chronic hepatitis B (CHB) (1.2%; 95% CI, 0.6%–2.2%), 16 with HBeAg-negative CHB (2.1%; 95% CI, 1.2%–3.3%) and 45 inactive HBV carriers (5.9%; 95% CI, 4.4%–7.8%). Sixty-one (8.0%; 95% CI, 6.2%–10.1%) patients showed OBI. Treated patients showed similar HBV DNA levels to those obtained in untreated patients, regardless of HBV patterns. Around 15.0% of OBI patients showed high (>1,000 UI/mL) viremia. The mutation M204V/I conferring resistance to 3TC was more common in HBV/A (47.4%) than in HBV/E isolates (0%) (*P* = .04). Our findings encouraged clinicians to promote HBV vaccination in patients with no exposure to HBV and to switch 3TC to universal TDF in those with CHB.

## Introduction

Hepatitis B virus (HBV) is associated with approximately 780,000 deaths each year worlwide, mostly due to the chronic course of hepatitis B infection [1]. HBV and human immunodeficiency virus type-1 (HIV-1) share common routes of transmission. To date, 35 million individuals worldwide are HIV-1 carriers, of which 3 to 6 million have chronic hepatitis B (CHB). HBV/HIV co-infection is a huge public health problem in sub-Saharan Africa where both viruses are endemic [2]. HBV-related end-stage liver disease, such as decompensated cirrhosis and hepatocellular carcinoma (HCC), represents a growing cause of morbidity and mortality, notably in patients with low CD4^+^T-cell counts and high HBV DNA levels[3].

In resource-limited settings (RLS), current guidelines recommend hepatitis B surface antigen (HBsAg) screening for all HIV-1-infected patients before antiretroviral treatment (ART) initiation, and the use of first-line ART regimens containing tenofovir (TDF) in patients co-infected with HIV and HBV [4, 5]. However, HBsAg testing is not systematically performed. For instance, despite the high prevalence rates of HIV (4.2%) [6] and HBV (12.9%) in Gabon [7], data on HIV/HBV co-infections are lacking for this country. Consequently, for many years, most African HIV-HBV co-infected patients received ART regimens containing 3TC alone that were sub-optimal and “HBV-blind” [8, 9]. In addition, many African HIV-infected patients who test positive for HBsAg receive no additional testing, such as hepatitis B e antigen (HBeAg) and/or HBV DNA viral load (VL) and total antibody to hepatitis B core antigen (HBcAb), which are appropriate markers for CHB and occult hepatitis B infection (OBI), respectively [10, 11]. HBV DNA levels are useful to differentiate between patients with HBV DNA ≥ 2,000 IU/mL, indicating likely HBeAg-negative CHB and inactive HBV carriers who have HBV DNA < 2,000 IU/mL [12].

HBV genotyping and the detection of specific clinically relevant HBV mutations have been seldom studied in African patients co-infected with HIV-1. To date, HBV is classified into 10 genotypes, designated HBV/A-J, with specific geographical distributions [13, 14]. Recently, genotype A has been subdivided into four subgenotypes named HBV/A1, HBV/A2, HBV/QS-A3 (quasi-species A3) and HBV/A4 [15]. HBV/QS-A3 has been reported almost exclusively in West and Central Africa. HBV/E is also highly endemic in West and Central Africa, and has low intra-genotype diversity suggesting its recent emergence [16]. Regarding HBV mutations, the use of 3TC alone has been shown to be strongly associated with consistently high HBV viremia due to the rapid emergence of HBV mutants carrying the M204I/V mutation conferring resistance to 3TC/FTC [17, 18]. Precore (PC) and basal core promoter (BCP) mutations may represent the predominant virus species in patients with HBeAg-negative CHB in many parts of the world [19]. Escape mutations in the “a” determinant of the major hydrophilic region (MHR) of HBsAg can impair the diagnostic performance of HBsAg assays and can lead to emergence of vaccine-escape mutants (VEM) and hepatitis B immunoglobulin (HBIG) therapy failure [20].

In this study, we assessed the serological and molecular HBV patterns, including genotyping and mutational analysis, in HIV-positive patients mostly treated with 3TC-based ART regimens without TDF in Gabon.

## Materials and Methods

### Patients

Our cross-sectional study was conducted among HIV-1 infected patients who attended a HIV care center located at Franceville (Southeastern Gabon) between March 2010 and January 2013. Whole blood specimens were collected by venipuncture (5 ml of EDTA-anticoagulated peripheral blood). Plasma samples were then stored at -80°C. Gender, age and ART were retrieved from patients’ files. CD4^+^ T-cell counts, HIV-1 RNA viral load (VL) measurements and HCV serology were performed in all patients at the time of sampling [21]. The study was approved by the Ethics Committee of Gabon (N°0021/2013/SG/CNE). Written informed consent was obtained from each patient.

### HBV serology

Total (IgM and IgG) HBcAb were first assessed using the Monolisa Anti-HBc PLUS immunoassay (ELISA) (Bio-Rad, Marnes-La-Coquette-France). In case of negative HBcAb results, subjects were then tested for antibody to HBsAg (HBsAb) titer with the Monolisa Anti-HBs PLUS ELISA (Bio-Rad). Subjects were considered as negative for HBsAb when titer was <10 mIU/mL. In case of positive HBcAb results, the detection of HBsAg was performed using the Monolisa HBs Ag ULTRA kit (Bio-Rad), a fully multivalent assay showing high sensitivity in the detection of HBV mutants [22]. If HBsAg result was negative, specimens were tested for HBsAb. All samples that tested positive for HBsAg were assessed for the presence of IgM HBcAb (Monolisa Anti-HBcIgM, Bio-Rad) and hepatitis B e antigen (HBeAg)/antibody to HBeAg (HBeAb) (Monolisa HBe Ag-Ab,Bio-Rad). All ELISA assays were performed according to manufacturer’s instructions.

### Quantitative real-time HBV DNA PCR

Using the Arrow extractor (NorDiag, Biotrin International, Ireland), DNA was extracted from 240 μl of plasma, pretreated with 10 μl of proteinase K, using the Arrow Viral NA extraction kit, according to the manufacturer’s instructions. Template DNA was eluted into 60 μl of kit elution buffer. Besides clinical samples, quantification standard (AcroMetrix HBV Panel, AcroMetrix, Menica, CA, USA) was also extracted for each run with the same protocol and 1:10 diluted (from 50,000,000 IU/mL to 50 IU/mL). For amplification, we used a primers/probe set designed under the auspices of “Agence Nationale de Recherches sur le SIDA et les hépatites virales” (ANRS 12187 project) and targeting a conserved region in the HBV S gene (nucleotide (nt) positions, 379–426). All runs were performed in a 50-μl volume containing DNA extract (10 μl), Master Mix (Platinum UGD, USA) (25 μl), pure water (HyClone Pure Water, Thermo Fisher Scientific, Waltham, MA USA) (12.5 μl), forward primHBV1 (5’-GTGTCTGCGGCGTTTTATCA-3’) and reverse primHBV2 (5’-AGGCATAGCAGCAGGATGAA-3’) primers at 10 μM (1 μl each) and probe (5’FAM-TGCGGCGTTTTATCAT-MGB3’) at 5 μM (0.5 μl). Each reaction consisted of: 2 min at 50°C and 10 min at 95°C; followed by 50 cycles of 15 sec at 95°C and 1 min at 60°C each.

The lower limit of quantification (LLOQ) of our technique was 100 IU/mL and the lower limit of detection (LLOD) was 50 IU/mL. In case of detected HBV DNA below LLOQ and above or equal to the LLOD, HBV DNA PCR was repeated once and if it was detected again, the mean value for duplicates was considered in the analysis. If not, samples were allocated a value equal to half the LLOD (25 IU/mL), as undetectable specimens (i.e, not amplified (n/a) samples with no value for cycle threshold (Ct)).

HBV DNA was quantified in specimens positive for HBsAg and in those with isolated HBcAb. In addition, 50 samples negative for both HBcAb and HBsAb were randomly selected for HBV DNA detection in order to evaluate the specificity of our real-time PCR assay.

### HBV genotyping and mutations by direct sequencing

Specimens positive for HBV DNA, with sufficient plasma, were studied for HBV genotyping and polymerase (*pol)* gene mutations in the HBV surface (S) gene. DNA extraction was done manually using the QIAamp Viral DNA Mini Kit (Qiagen, Courtaboeuf, France) and required 200 μl of plasma. HBV was amplified using a semi-nested PCR for the HBV S gene (nt positions, 56 to 911), as previously published by Olinger et al. [23]. In addition, PC and BCP regions were amplified with a nested PCR (nt positions, 1672 to 2012) that targets a fragment of 340 bp in the core (C) gene, as previously published [24].

Sequencing was performed on the 3730XL DNA Analyzer (Applied Biosystems, Vernon Hills, Illinois, USA). Pairwise alignments were performed using ClustalW (1.6) model with sequences available in GenBank, and representing all HBV genotypes. [25, 26]

Phylogenetic analyses was carried out by Bayesian inference using the Bayesian Markov chain Monte Carlo (MCMC) statistical framework implemented in MrBayes version 3.1.2 software [27, 28] under the model ‘general reversible model of evolution’ with a gamma distribution of rates across sites, run for 8 x 10^6^ generations, with a burn-in of 25%. Calculations of Effective Sample Size (ESS) were examined with the TRACER v1.5 program (http://evolve.zoo.ox.ac.uk/) and all the ESS were greater than 1200. The tree datasets were computed using strict consensus and majority rules algorithms to evaluate the posterior probabilities of branching pattern (BPP). The final tree was visualized in the FigTree version 1.4.2 (program 2006–2014, Andrew Rambaut Institute of Evolutionary Biology, University of Edinburgh. http://tree.bio.ed.ac.uk/).

Using the Mutation Reporter Tool software (http://hvdr.bioinf.wits.ac.za/mrt/), HBV resistance-associated mutations (RAMs) in the *pol* gene represented by V173L, L180M, A181V, A194T, S202G, M204V/I and N236T were assessed [29] [30, 31]. In addition, we looked for VEMs outside (at amino-acid (aa) position 120) and within the HBsAg immunodominant ‘a’ determinant (at aa positions 126, 129, 130, 144 and 145) [32]. Finally, A1762T/G1764A mutations in BCP region and T1858C, G1862C andG1896A mutations in PC region were analyzed [33].

### Statistical analysis

Prevalence was expressed with 95% confidence intervals (95% CI). Continuous data were expressed as median value with 1^st^ and 3^rd^ interquartile ranges (IQR). Comparisons between groups were conducted using the Mann-Whitney test, while categorical data were expressed as percentages and compared across groups using the χ2 test. Results were 2-sided and *P* values < 0.05 were considered to be statistically significant.

## Results

### Characteristics of Study Population

A total of 762 HIV-1-positive patients were studied. Among these, 560 (73.5%) were women. Median age was 41.5 years (IQR, 34.7–49.0 years). Among the 507 treated patients, ninety-eight percent was receiving 3TC-based regimens without TDF. Median time on ART was 36 months (IQR, 13–57 months). Overall, 52.1% (264/507) of treated patients showed undetectable HIV-1 RNA VL results (Table 1).

**Table 1:** Main characteristics of patients co-infected with HIV-1 and HBV in Franceville, Gabon (2010–2013).

Characteristics	Total	Male	Female
**Patients, n (%)**	762 (100)	202 (26.5)	560 (73.5)
**Age, median years (IQR)**	41.5 (34.7–49.0)	45.0 (39.4–52.1)	39.3 (33.2–48.0)
**ART**			
No, n (%)	255 (33.5)	75 (37.1)	180 (32.1)
Yes, n (%)	507 (66.5)	127 (62.9)	380 (67.9)
**Type of ART**			
3TC-based regimen (without TDF), n (%)	497 (98)	120 (94.5)	377 (99.2)
TDF-based regimen, n (%)	10 (2)	7 (5.5)	3 (0.8)
**Duration on ART, months (IQR)**	36 (13–57)	30 (9–56)	38 (17–57)
**CD4 count, median cells/ml (IQR)**			
Untreated patients	382 (162–618)	355 (201–512)	467 (245–636)
Treated patients	344 (187–487)	307 (174–462)	352 (192–499)
**HIV-1 RNA, median Log**_**10**_ **copies/mL (IQR)**			
Untreated patients	4.93 (4.42–5.72)	5.26 (4.77–6.08)	4.86 (4.04–5.68)
Treated patients	< 1.24 (< 1.24–4.87)	< 1.24 (<1.24–4.86)	< 1.24 (<1.24–4.89)
**Positive HCV serology result**, n (%)	56 (7.3)	16 (7.9)	40 (7.1)

Abbreviations: ART, antiretroviral treatment; 3TC, lamivudine; TDF, tenofovir; IQR, inter quartile range.

### HBV patterns among HIV-1-infected patients

**Fig 1:**
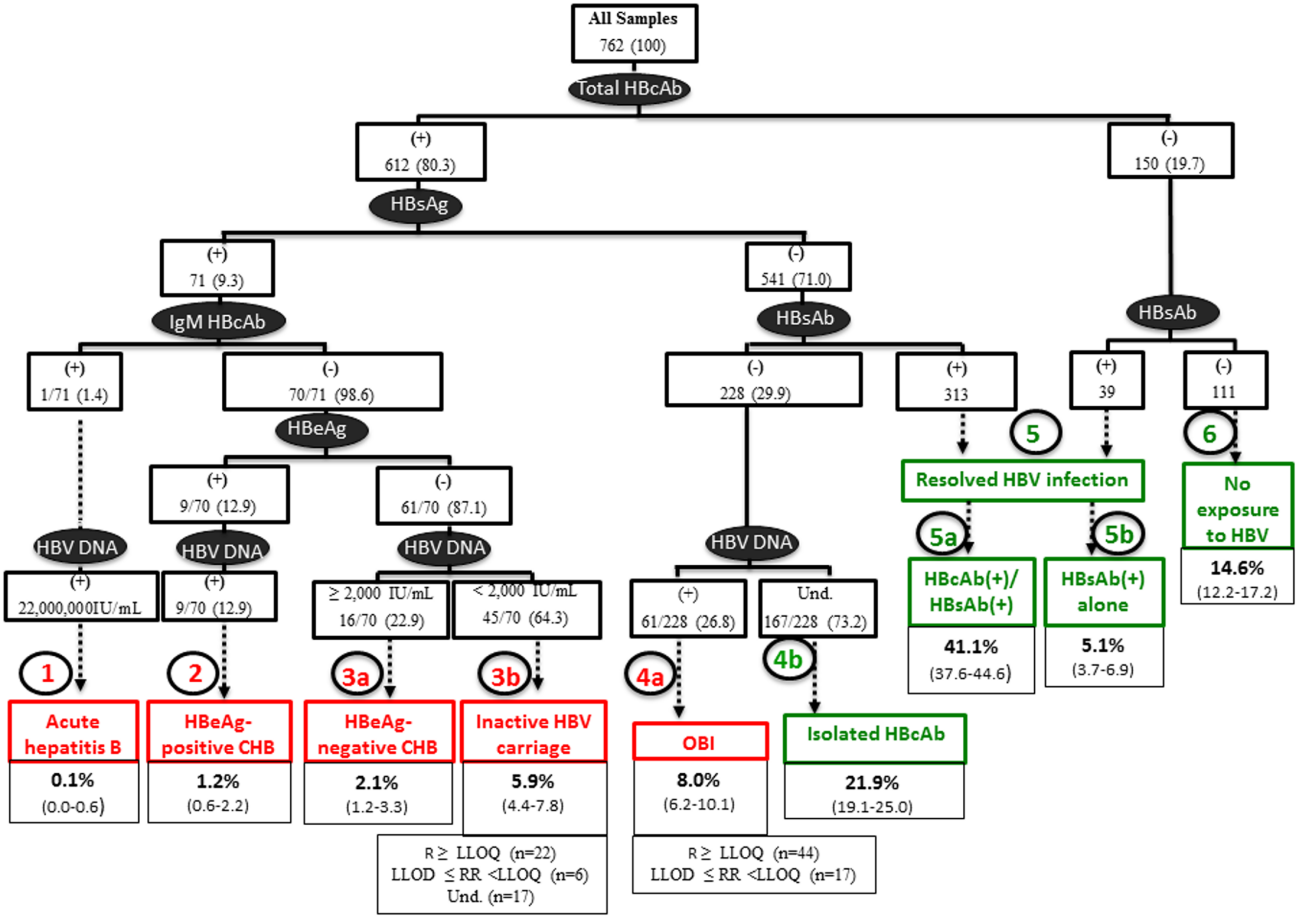
HBV patterns defined by serological results and serum HBV DNA levels. Infectious patterns are indicated in red boxes. Non-infectious patterns are indicated in green boxes. Overall prevalence rates are n (%) unless otherwise stated. Abbreviations: (+), positive; (-), negative; Und., undetectable; R, reactive; RR, repeatedly reactive; CHB, chronic hepatitis B; OBI, hepatitis B occult.

As summarized in Fig 1, nine distinct profiles were identified. First, among the 71 patients found positive for total HBcAb and HBsAg, one (0.1%) only tested positive for anti-HBc IgM (acute hepatitis B, profile 1) and had a high VL result (22,000,000 IU/mL). Second, among the 70 remaining HBc IgM-negative patients, nine (12.9%) tested positive for HBeAg with detectable HBV DNA levels (HBeAg-positive CHB, profile 2), representing 12% of the total population. Next, 61 (87.1%) tested negative for HBeAg; among which, 16 (2.1%) showed HBV DNA results ≥ 2,000 IU/mL (HBeAg-negative CHB, profile 3a) whereas 45 (5.9%) had HBV DNA values < 2,000 IU/mL (inactive HBV carriers, profile 3b). Among the 541 patients found positive for total HBcAb but negative for HBsAg, 228 tested negative for HBsAb, including 61 (8.0%) patients showing detectable DNA levels (OBI, profile 4a) *versus* 167 (21.9%) with undetectable HBV DNA (isolated HBcAb profile, profile 4b). Next, 313 (41.1%) individuals were positive for total HBcAb and HBsAb (resolved HBV infection, profile 5a). In the 150 patients who tested negative for total HBcAb, 39 (5.1%) had HBsAb (profile 5b) and 111 had no marker for HBV (no exposure to HBV,profile 6). HBV DNA was not amplified in 50 samples taken from patients with no exposure to HBV. No significant difference was observed between patients belonging to profiles 2, 3 and 4, according to gender, CD4 counts, HIV-1 RNA levels and HCV Ab positivity (data not shown).

**Fig 2:**
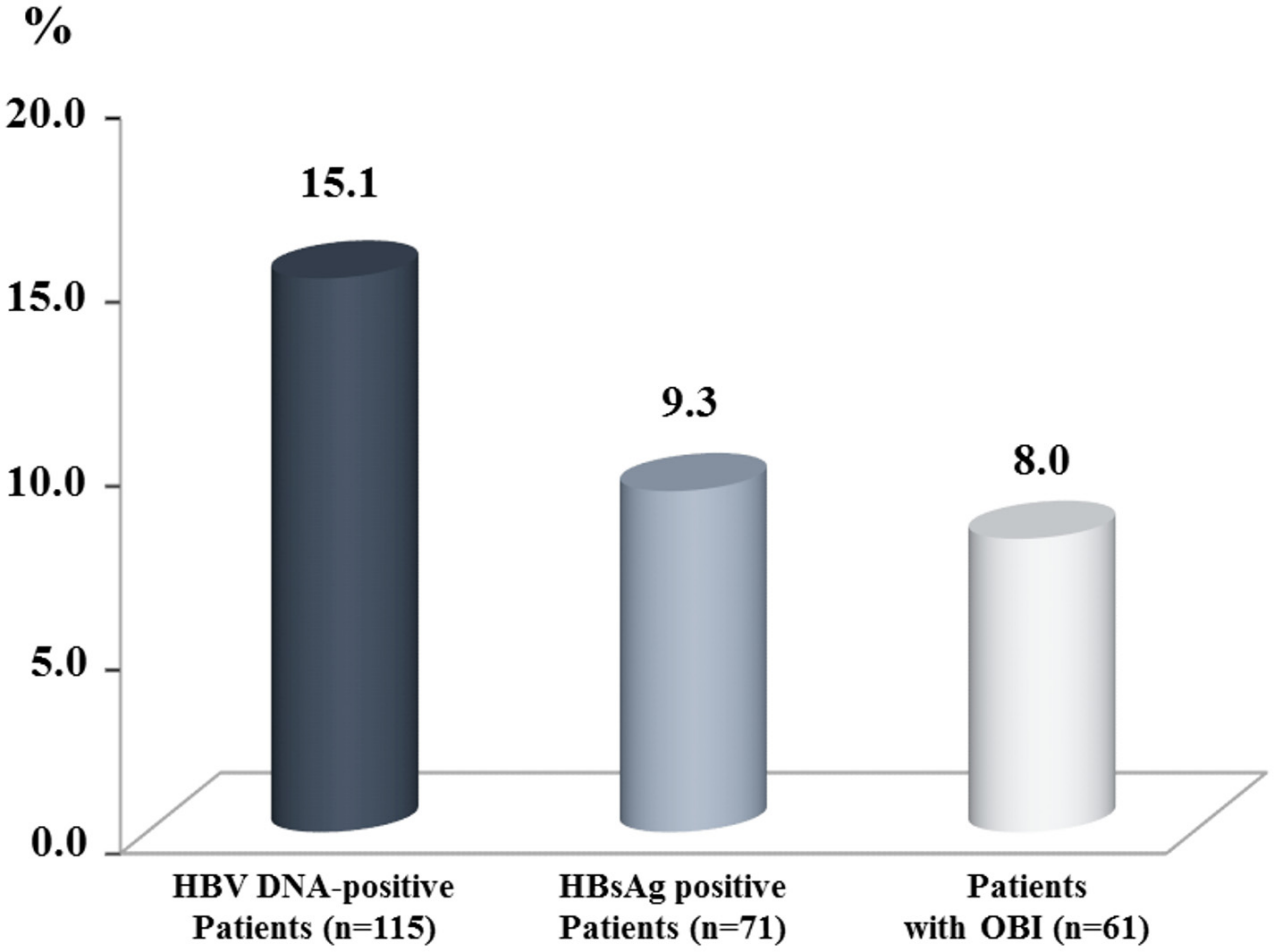
Prevalence rates of patients with detectable HBV DNA results, patients with overt (presence of circulating HBsAg) HBV infection (acute or chronic), and patients with OBI.

**Fig 3:**
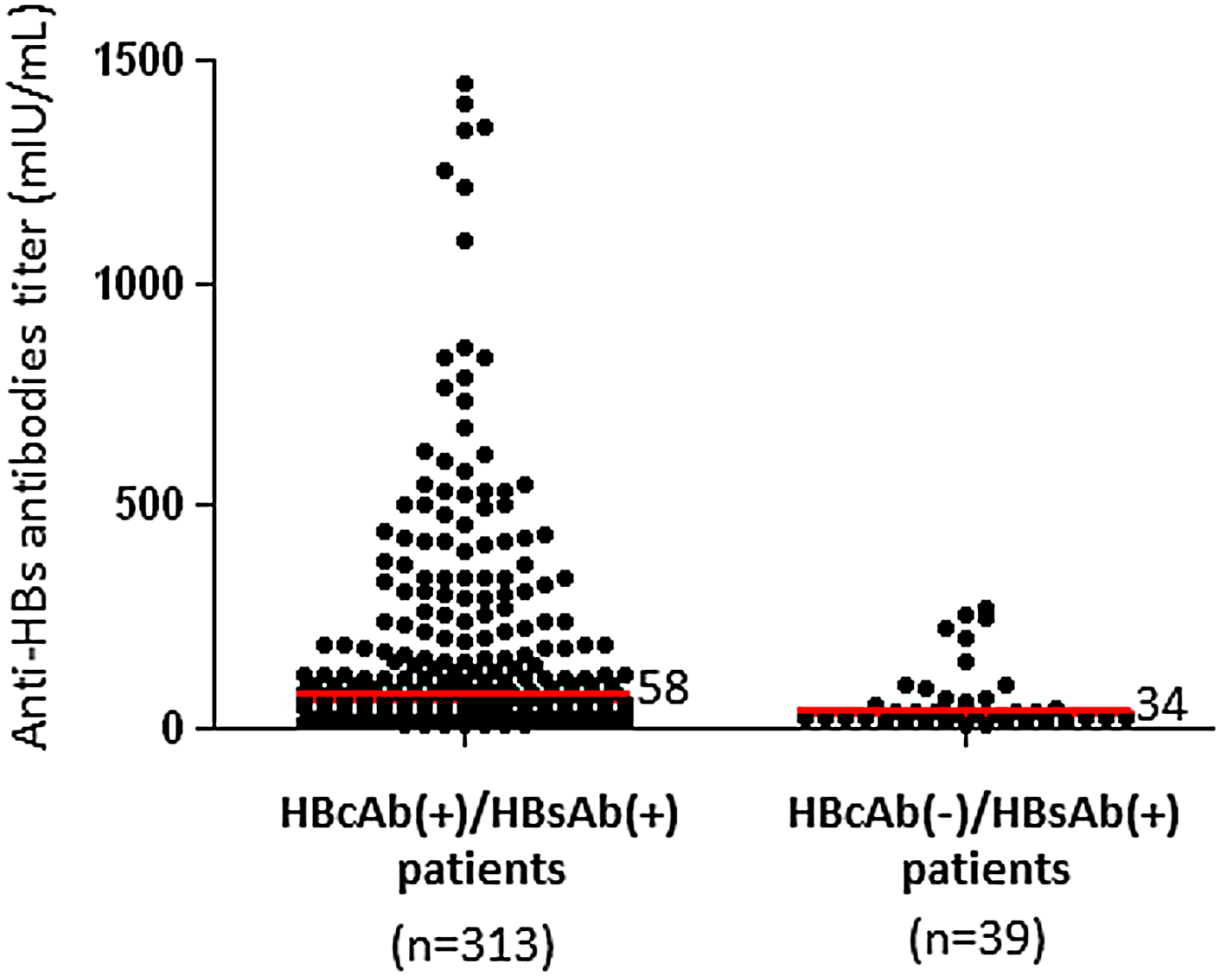
Comparison of anti-HBs antibodies levels in patients positive for both anti-HBc and anti-HBs Ab (profile 5a) and those with only anti-HBs Ab (profile 5b).

Overall, the prevalence of OBI (61/762, 8.0%) was similar to that obtained for overt (presence of circulating HBsAg) HBV infection (71/762, 9.3%) (*P* = .36) (Fig 2). The median anti-HBS Ab titer in patients belonging to profile 5a was significantly higher (58 mIU/mL, IQR, 30–182) than that obtained in patients belonging to profile 5b (34 mUI/mL, IQR, 19–67) (*P* = .0008) (Fig 3).

### Impact of antiretroviral treatment on HBV DNA levels according to HBV patterns

**Fig 4:**
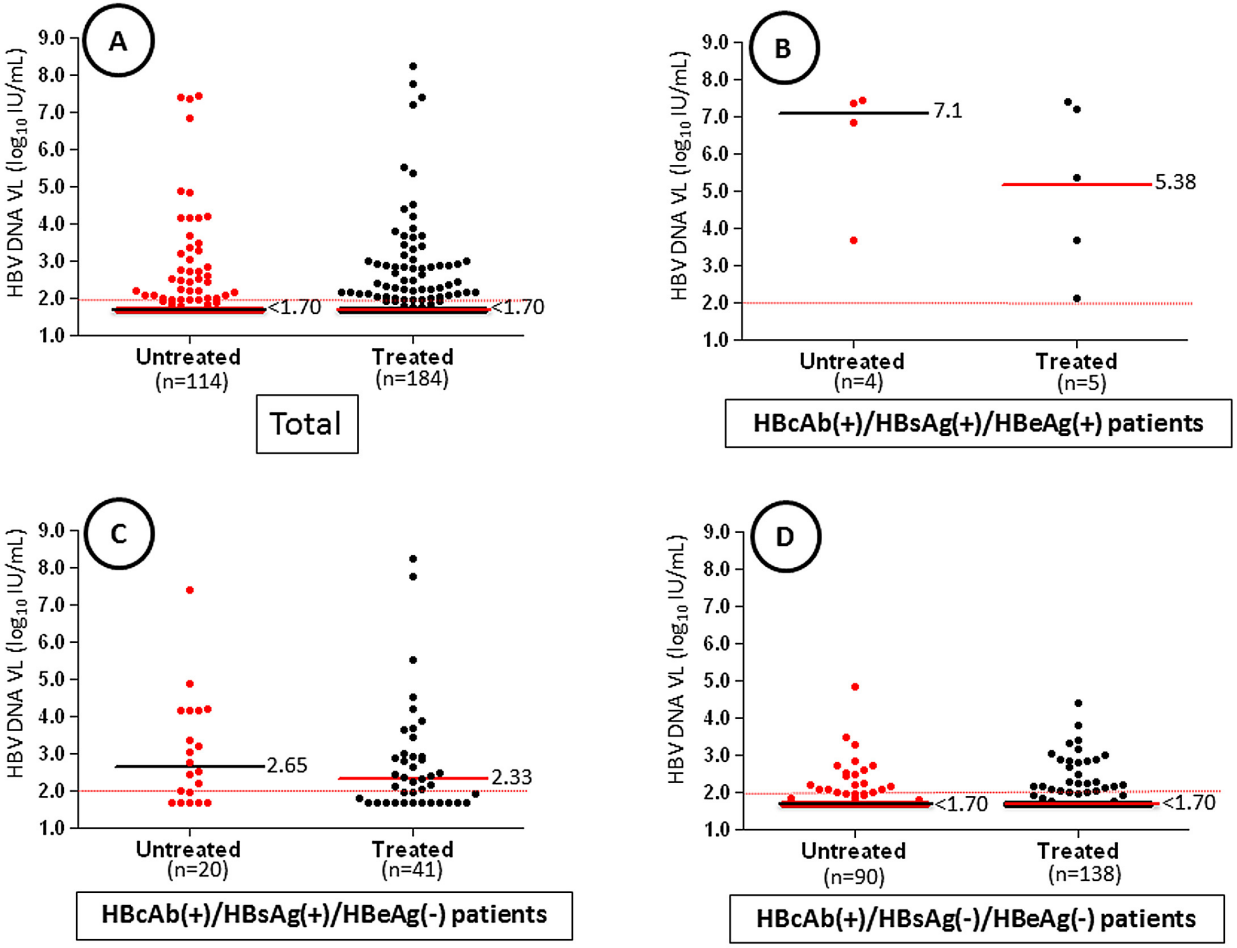
Comparison of HBV DNA levels in 298 patients co-infected with HIV-1. **A)** Overall comparison between patients treated with ART versus untreated patients. **B)** Comparison in HBsAg-positive patients with HBeAg-positive chronic hepatitis B (profile 2). **C)** Comparison in HBsAg-positive patients with HBeAg-negative pattern (profile 3). **D)** Comparison in patients with OBI or isolated anti-HBc antibodies (profile 4). Median HBV DNA levels are indicated with black bars for untreated patients and with red bars for treated patients. The red dotted line indicates the limit of detection (LOD) of the assay.

Overall, median HBV DNA levels were similar in both untreated and treated patients (*P* = 0.64) (Fig 4A). The lack of significance between untreated and treated patients was obtained, regardless of HBV patterns: 7.1 versus 5.38 log_10_ IU/mL in profile 2 (*P* = .41) (Fig 4B); 2.65 versus 2.33 log_10_ IU/mL in profile 3 (*P* = .34) (Fig 4C); and <1.70 versus < 1.70 log_10_ IU/mL in profile 4 (*P* = .59) (Fig 4D). Among the 61 patients with OBI, nine (14.7%) had HBV DNA level > 1,000 IU/mL, 35 (57.4%) had HBV DNA level between 100–1,000 IU/mL and 17 (27.9%) had repeatedly reactive HBV DNA results below the limit of detection.

### Distribution of HBV genotypes

HBV genotype was successfully analyzed in 28 samples (on 114 specimens with HBV DNA levels ≥ 50 IU/mL, 26%) from patients with HBeAg-positive CHB (n = 3), negative-HBeAg CHB (n = 6), inactive HBV carriage state (n = 9) and reactivated OBI (n = 10). PCR failed from 22 (19.3%) samples whereas 64 (56.1%) had insufficient volumes of plasma. All sequences described herein are available in the GenBank (accession numbers KM983561 to KM983588).

**Fig 5:**
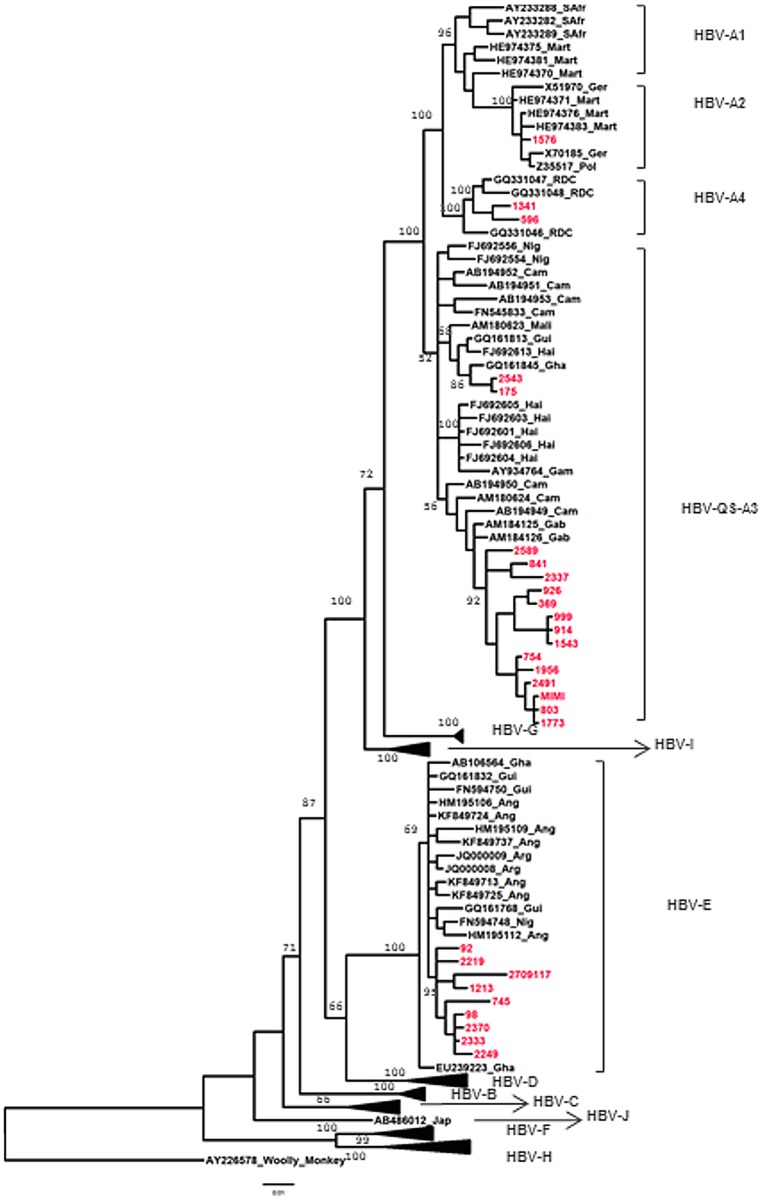
Phylogenetic analysis of a 765 bp HBV-S gene region from different HBV isolates, with Woolly Monkey strain (AY226578) as the root. The phylogenetic tree was inferred by the Bayesian method in the GTR model, with gamma-distributed rates at sites, 8 million generations and effective sample sizes greater than 1200. BPP (Branching Posterior Probability) are shown along the main nodes. HBV sequences obtained during this study are represented in red.

As shown in Fig 5, we revealed the presence of two HBV genotypes, including 19 sequences (67.9%) clustering with genotype HBV/A and nine (32.1%) with genotype HBV/E. For genotype A, most (n = 16) strains clustered with sub-genotype QS-A3, and were closely related to isolates from Cameroon and Gabon. Two additional strains were classified as sub-genotype A4 and one clustered with sub-genotype A2. The nine HBV/E strains were closely related to isolates from Angola and Ghana. Genotype HBV/A was more frequently encountered in females (14/19, 73.7%), whereas HBV/E was found in a majority of males (7/9, 77.8%) (*P* = .03).

### Distribution of specific HBV mutations according to HBV genotype

**Fig 6:**
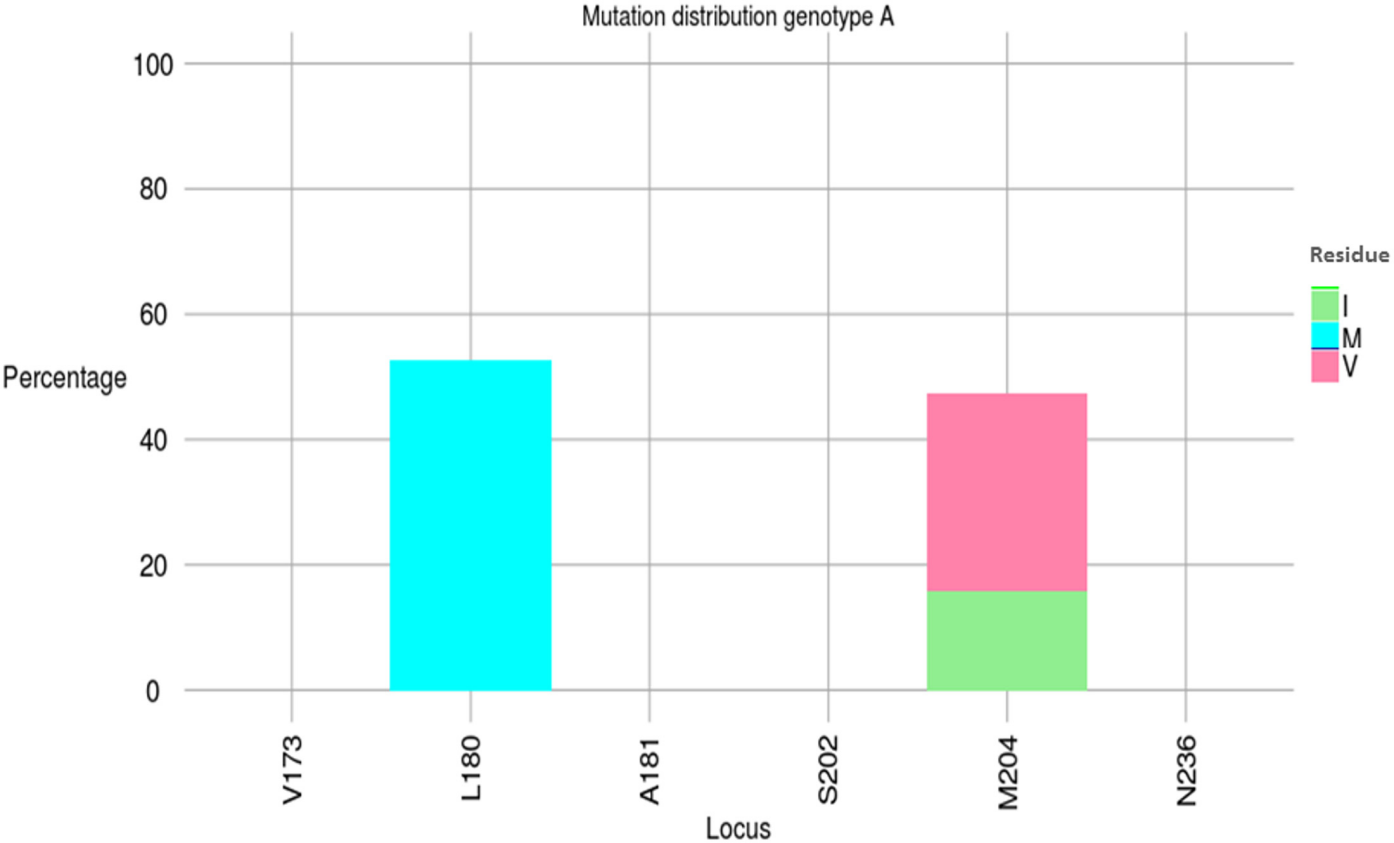
Mutation distribution graphs for the polymerase region. Sequence alignments were submitted to Mutation Reporter Tool (http://hvdr.bioinf.wits.ac.za/mrt/) to produce graphs for genotype A. None mutation found in nine sequences from genotype E. Letters preceding each locus on the X-axis show the reference motifs. To facilitate comparison, conserved loci were not suppressed.

Regarding RAMs, the mutation M204V/I, associated with the compensatory mutation L180M, was significantly more common in genotype HBV/A (9/19, 47.4%) than in genotype HBV/E (0/9, 0%) (Yate’s corrected χ2, *P* = .04) (Fig 6, Table 2). The median duration on 3TC-based ART was not significantly different between patients harboring HBV/A strains (21 months; IQR, 0–55.5) and those carrying HBV/E strains (5.5 months; IQR, 0–49.5) (*P* = .66). Among the 9 patients harboring HBV/A variants with RAMs, six were treated with 3TC-based ART whereas three were untreated.

**Table 2:** Specific HBV mutations according to HBV patterns and genotypes.

ID	Sex	Age (yrs)	CD4 (/mL)	HIV-1 RNA (log)	ART duration (months)	HBV						
						HBV DNA (log)	Pattern	GT	Viral mutations			
									RAMs	VEM	BCP	PC
175	M	51	54	6.02	0	7.45	2	A	WT	WT	A1762T/G1764A	WT
329	F	40	499	3.19	60*	7.39	2	A	L180M, M204V	WT	WT	WT
2219	M	58	94	4.66	0	7.37	2	E	WT	WT	WT	WT
2709117	M	45	10	5.52	0	7.40	3a	E	WT	WT	WT	G1896A
MIMI	F	50	618	<1.24	4*	5.52	3a	A	WT	T126I	WT	G1896A
841	F	46	752	<1.24	98*	4.52	3a	A	WT	WT	WT	WT
2337	M	42	34	6.45	0	4.17	3a	A	WT	WT	WT	WT
2370	F	53	426	4.44	0	4.15	3a	E	WT	WT	A1762T/G1764A	G1896A
1213	M	40	666	3.42	67*	3.44	3a	E	WT	WT	nd	nd
2249	M	46	37	4.40	0	3.06	3b	E	WT	P120T	WT	WT
1341	F	36	618	<1.24	34*	3.02	3b	A	WT	WT	nd	nd
2543	M	40	266	<1.24	6**	2.94	3b	A	WT	WT	nd	nd
1543	F	49	318	5.21	30*	2.79	3b	A	L180M, M204V	WT	nd	nd
914	F	38	618	3.89	12*	2.52	3b	A	L180M, M204V	WT	nd	nd
2589	F	34	405	4.0	4**	2.47	3b	A	WT	WT	nd	nd
596	F	31	227	<1.24	53*	2.43	3b	A	WT	WT	WT	WT
2333	M	46	372	<1.24	11*	2.39	3b	E	WT	WT	A1762T/G1764A	G1896A
999	F	33	970	<1.24	58*	2.03	3b	A	L180M, M204V	WT	WT	WT
1956	F	30	258	nd	0	4.84	4a	A	L180M, M204V	WT	nd	nd
98	F	50	207	6.41	85*	4.41	4a	E	WT	WT	A1762T/G1764A	G1896A
1773	F	38	533	<1.24	21*	3.82	4a	A	L180M, M204I	WT	nd	nd
1576	M	35	533	3.09	0	3.50	4a	A	WT	WT	WT	G1896A
803	M	44	208	5.59	46*	2.80	4a	A	L180M/M204I	WT	nd	nd
2491	F	29	277	4.90	0	2.73	4a	A	L180M/M204I	WT	nd	nd
926	F	70	488	3.56	0	2.72	4a	A	L180M/M204V	WT	nd	nd
754	F	37	22	5.07	81*	2.69	4a	A	WT	WT	nd	nd
92	M	46	316	3.84	19**	2.63	4a	E	WT	WT	nd	nd
745	M	40	547	<1.24	32*	2.63	4a	E	WT	WT	nd	nd

*3TC-based ART;

**TDF-based ART.

Abbreviations: ART, antiretroviral treatment; F, female; M, male; GT, genotype; BCP, basal core promoter; PC, precore; WT, wild type; nd, not done.

Regarding influence of mutations in the polymerase on the S-gene, no mutation implicated in vaccine escape was found in all genotypes.

The BCP/PC region was successfully sequenced in 14 strains (failed PCR in five samples and insufficient volumes in nine). One HBV/A strain (1/8, 12.5%) had BCP mutations whereas three (3/6, 50%) HBV/E strains showed the A1762T and G1764A double mutation (*P* = .35) (Table 2). The classical PC G1896A mutation was found in two (2/8, 14%) and four (4/6, 66.7%) HBV/A and HBV/E isolates, respectively (*P* = .31). All 14 sequences are available in the GenBank (accession numbers KT724763 to KT724776).

## Discussion

Our study provided a comprehensive picture of HBV patterns identified in a large group of HIV-1-positive patients in Gabon who were mostly receiving 3TC-containing ART without TDF. We found herein prevalence rates of HBeAg-positive CHB, HBeAg-negative CHB, inactive HBV carriage and OBI of 1.2%, 2.1%, 5.9% and 8.0%, respectively. In our relatively old population, HBsAg-positive patients in the HBeAg-negative phase were approximately 6 times more frequent than those who were in the HBeAg-positive window, likely reflecting the natural history of HBV infection in sub-Saharan Africa where most individuals are infected at childhood by horizontal transmission [34].

As recommended by WHO, our survey supports the universal use of TDF because no significant difference was obtained in HBV DNA levels between patients receiving 3TC-containing regimens versus untreated individuals, regardless of HBV patterns. When considering HBV/A genotypes only, we observed a high (~50%) prevalence of M204V/I and L180M mutations. Our data are consistent with those obtained in Malawi reporting HBV DNA VL rebounds coinciding with the rapid emergence of major resistance and compensatory mutations coexisting on the same HBV genomes [9]. In another study conducted in Cameroon, RAMs were detected in 60% of HIV/HBV co-infected patients at month 24 [18]. Importantly, we identified in our survey three natural M204V/I mutations in 3TC-naive patients, as already described in other settings [17]. For these three patients, we were not able to identify any previous 3TC-based treatment. Taken together, these findings highlight the urgency to abandon the use of 3TC as the sole HBV active agent and to switch to TDF in HIV/HBV co-infected patients.

Consistently with other studies conducted either in Africa [10, 35] or Asia [36], HBcAb was a reliable surrogate marker of OBI. Unexpectedly, among our patients with HBcAb ‘alone’, ~20% (44/228) showed HBV DNA levels above 100 IU/mL. Previous studies assessing HBV viremia in subjects with HBcAb alone have reported widely divergent results. For instance, Launay and colleagues reported HBV viremia exceeding 10^4^ copies/mL in six of ten patients with OBI [37]. In contrast, Khamduang *et al*. obtained three (1.5%) specimens with HBV DNA levels between 101 and 1,000 IU/mL among 200 Thai HIV-positive pregnant women with OBI [36]. It is likely that high HBV DNA levels in OBI are more common during routine HIV care in RLS where unplanned 3TC-based ART interruptions are highly prevalent (>40% in our population, as previously documented) [6], and are recognized to be associated with severe HBV reactivations [38–40].

Our study revealed a highly complex molecular virology of HBV during HIV co-infection. First, a dual QS-A3 and E HBV genotypic presence was confirmed in HIV-positive patients [7, 41], suggesting that mixed infections and/or recombination events are possible in Gabon. Second, we describe for the first time in the S gene, in this country, the presence of sub-genotypes A2 and A4, highlighting the high degree of HBV/A genetic diversity in Africa. However HBV full-genome sequences are required in our country to identify recombinant A/E strains and confirm the circulation of HBV sub-genotype A2 and A4, there origin and how they integrated this country. Third, the M204V/I RAM were more common in HBV/A strains. Fourth, PC/BCP mutants were also prevalent, notably in HBV/E genotypes. Our findings suggest that HBV genotypes should be further monitored, at least in African research laboratories, because they may display specific mutational S, P and C patterns that can differentially impact on HBV treatment and prevention.

Our study has limitations, including its cross-sectional design and the absence of alanine aminotransferase (ALT) values. One important limitation was represented by the loss of significant information on HBV genotypes and mutations since we were able to analyze only 26% of HBV DNA-positive patients. It was mostly due to the unavailability of relevant plasma specimens. It made difficult the identification of the most common mutations in HBeAg-negative patients in our setting. Another strong limitation of our study was represented by OBI assessment. At the time of our survey, OBI was defined by the presence of isolated anti-HBc with detectable (<200 IU/mL) serum HBV DNA. To date, according to the last statements (Taormina definition), the diagnosis of OBI requires detection of DNA positivity in at least two different genomic regions (to avoid the overestimation of OBI due to DNA false positivity), with a sensitivity of detection of <10 copies of HBV DNA per reaction (to prevent the underestimation of OBI). However, our assay constituted a unique opportunity to quantify HBV DNA in a RLS like ours because it was specific, cheap (~25–30 USD per a test) and could be performed on an open platform that was already used for HIV-1 RNA monitoring. On the other hand, the bases for the failure of HBsAg detection, despite high (>10^3^−10^4^ copies/mL) HBV DNA levels and no apparent immune escape mutations, remain obscure. To explain this striking pattern, different hypothesis (such as the significant impact of HBV/E on the sensitivity of HBsAg assays, or the presence of HBV surface gene mutations located outside the sequenced region) have been previously put forward by others (Launay et al, 2011). Finally, the clinical relevance of HBV DNA levels, HBV genotypes and HBV mutations among HIV/HBV co-infected patients was not investigated according to HBV-related liver disease.

**Fig 7:**
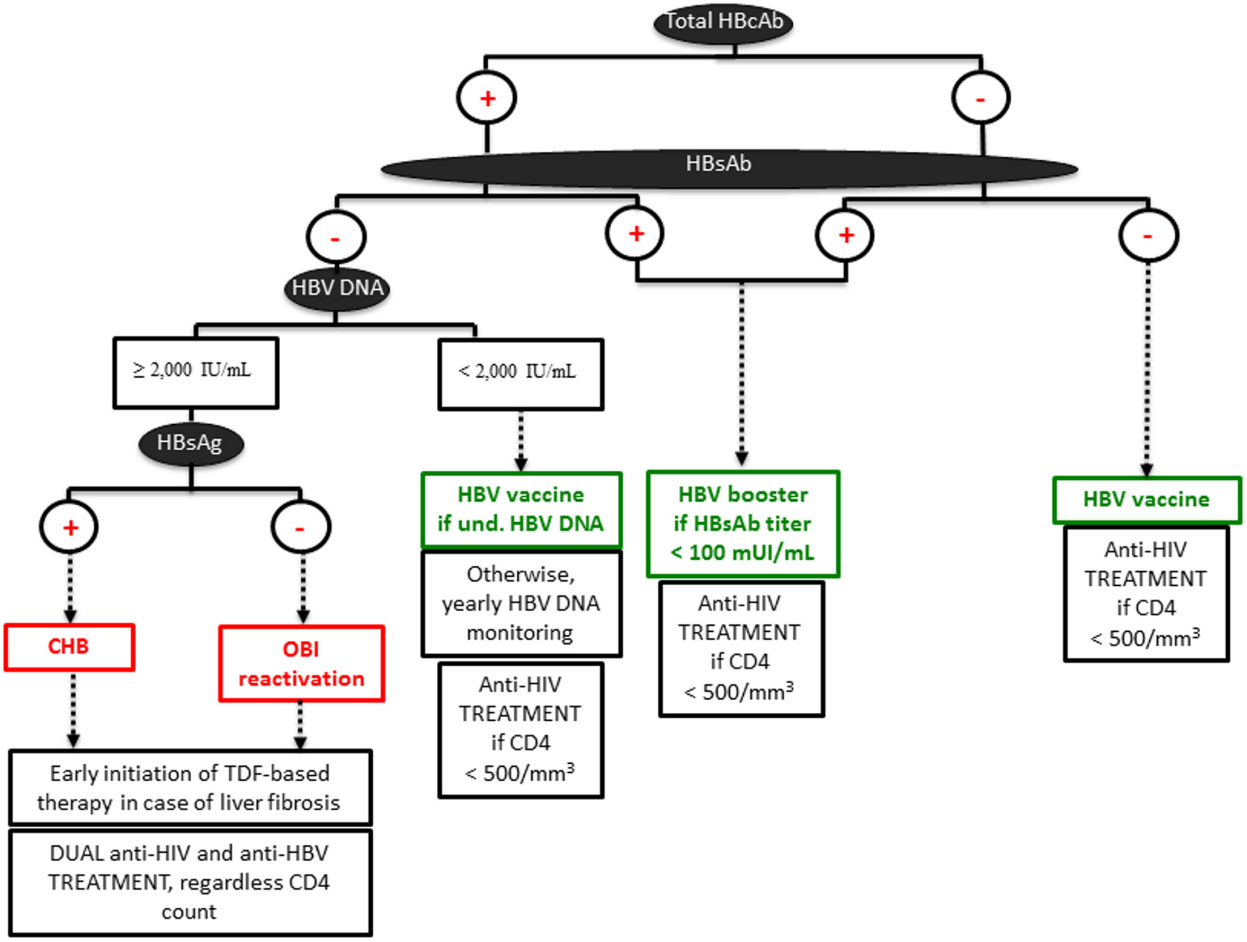
Suggested diagnostic strategy for HBV screening in resource-limited settings.

In conclusion, besides HCV [21] and HDV [7, 41], which are also highly prevalent in Gabon, we propose a new standardized diagnostic procedure for HBV screening in HIV-positive people from sub-Saharan Africa, before ART initiation (Fig 7). In this algorithm, initial testing should be done using total HBcAb, and HBsAb allowing HBV vaccine to be further implemented in patients with no exposure to HBV [42]. In addition, a booster vaccine dose may be offered in HBsAb(+) and HBcAb(+)/HBsAb(+) patients showing low (<100 IU/mL) HBsAb levels. Gabon introduced hepatitis B in its universal infant Expanded Programme of Immunization (EPI) in 2004 [43], indicating that patients in the present study were likely not immunized due to HBV vaccine but through HBV resolved infections. Patients who are found positive only for HBcAb should have an initial assessment for HBV DNA VL. If HBV DNA level is < 2,000 IU/mL, a simple VL follow-up is required. HBV vaccine should be promoted in case of undetectable VL results. In contrast, if HBV DNA VL result is ≥ 2,000 IU/mL, HBsAg should be done to differentiate patients with CHB and those with OBI activation. A dual, TDF-based, anti-HIV and anti-HBV therapy will be then initiated in these patients in case of liver fibrosis. Patients treated with 3TC will switch to TDF since prior exposure and/or resistance to 3TC does not compromise TDF-based regimens efficiency [44].
